# Anthropogenic food provisioning and immune phenotype: Association among supplemental food, body condition, and immunological parameters in urban environments

**DOI:** 10.1002/ece3.3814

**Published:** 2018-02-17

**Authors:** Jusun Hwang, Yongbaek Kim, Sang‐Won Lee, Na‐Yon Kim, Myung‐Sun Chun, Hang Lee, Nicole Gottdenker

**Affiliations:** ^1^ Department of Veterinary Pathology College of Veterinary Medicine University of Georgia Athens GA USA; ^2^ College of Veterinary Medicine Seoul National University Seoul Korea; ^3^ College of Veterinary Medicine Kon‐Kuk University Seoul Korea

**Keywords:** bacterial killing assay, IgG concentration, immune function, stray cat, supplemental feeding, urban habitat

## Abstract

Direct or indirect supplemental feeding of free‐ranging animals occurs worldwide, resulting in significant impacts on population density or altered demographic processes. Another potential impact of increased energy intake from supplemental feeding is altered immunocompetence. As immune system maintenance is energetically costly, there may be trade‐offs between immune responses and other energy‐demanding physiological processes in individual animals. Although increased availability of food sources through supplemental feeding is expected to increase the overall immunocompetence of animals, empirical data verifying the association between supplemental feeding and different immune parameters are lacking. Understanding the potential influence of supplemental feeding on immune phenotypes is critical, as it may also impact host–pathogen dynamics in free‐ranging animals. Using urban stray cats as a study model, we tested for associations between the intensity of supplemental feeding due to cat caretaker activity (CCA); body condition; and immune phenotype (bacterial killing assay (BKA), immunoglobulin G (IgG) concentration, and leukocyte counts). Significantly higher bacterial killing ability was observed in cats from high CCA districts, whereas higher IgG concentration and eosinophil counts were observed in cats from low CCA districts. Other leukocyte counts and body condition indices showed no significant association with CCA. We observed varying patterns of different immune components in relation to supplemental feeding. Out data suggest that supplemental feeding influences immune phenotype, not only by means of energy provisioning, but also by potentially reducing exposure rates to parasite infections through stray cat behavioral changes.

## 1 INTRODUCTION

Host immunity plays a critical role in regulating parasite infections (Downs, Adelman, & Demas, 2014; Hawley & Altizer, 2011). Immune response activation is energetically costly and may impact various life history traits (Downs & Stewart, 2014; Downs et al., 2014; Martin, Scheuerlein, & Wikelski, 2003) through energy trade‐offs and resource allocation (Ardia, Parmentier, & Vogel, 2011; French, Moore, & Demas, 2009). Reduction in immune responses in exchange for other energy‐demanding processes, such as juvenile growth, molting, or reproduction (Ardia, 2005; Landete‐Castillejos, García, Gómez, Laborda, & Gallego, 2002; Moreno‐Rueda, 2010; Nordling, Andersson, Zohari, & Gustafsson, 1998) can further affect host–parasite interactions by increasing the susceptibility of animals to parasite infections (French, DeNardo, Greives, Strand, & Demas, 2010; Lifjeld, Dunn, & Whittingham, 2002; Nordling et al., 1998; Sheldon & Verhulst, 1996). Trade‐offs between immune responses and other physiological processes are determined by multiple factors, such as availability of food sources (Downs et al., 2014; French & Moore, 2008; Martin, Navara, Weil, & Nelson, 2007). For instance, vitellogenic female tree lizards (*Urosaurus ornatus*) had significantly lowered wound‐healing rates, but captive vitellogenic lizards with ad libitum food access did not show this immune function‐related trade‐off (French & Moore, 2008; French et al., 2009). Positive associations between higher resource quality or resource availability and increased immune responses have been observed in both experimental and observational studies (Martin et al., 2007, 2008; Wilcoxen et al., 2015), emphasizing the role that sufficient energy intake plays in maintaining immune function.

In this context, supplemental feeding of wildlife may also affect immune responses of free‐ranging animals (Becker, Streicker, & Altizer, 2015; Brzek & Konarzewski, 2007). Human food subsidies can provide additional energy sources for wildlife that minimize trade‐offs between immune responses and other energetically costly physiological processes (Downs et al., 2014; Oro, Genovart, Tavecchia, Fowler, & Martínez‐Abraín, 2013). Although positive effects of supplemental feeding on body condition have been observed (Auman, Meathrel, & Richardson, 2008; Cypher & Frost, 1999; Jessop, Smissen, Scheelings, & Dempster, 2012; Otali & Gilchrist, 2004), few studies explore interactions between supplemental feeding, body condition, and immune responses (Forbes et al., 2016; Ruiz, French, Demas, & Martins, 2010; Wilcoxen et al., 2015). Nevertheless, epidemiological models suggest that immune defense responses to supplemental feeding are important drivers of infectious disease dynamics in wildlife populations (Becker & Hall, 2014). Such findings motivate examination of associations among food resources, host condition, and immune function in urbanized wildlife exposed to human food provisioning.

The objective of this study was to investigate potential associations between supplemental feeding, body condition, and immune phenotype in urban stray cats by examining multiple immune parameters and body condition indices. Our study system consists of stray cat populations in Seoul, Korea, a highly urbanized metropolitan city. These cats are exposed to different intensities of “cat caretaker activity” that primarily consists of supplemental food provisioning (Kim, Hwang, Min, Chun, & Lee, 2016). We hypothesize that stray cats from districts with high CCA will show higher body condition and immunocompetence measurements than cats from districts with lower CCA. Increasing populations of cats inhabiting in urban neighborhoods are known to frequently interact with city residents through various routes, potentially exposing each other to interspecies pathogen transmission, including feline‐borne zoonotic pathogens, either through direct (regular feeding, amicable interaction) or through indirect (e.g., defecation in playground sand) contacts (Schmidt, Lopez & Collier 2007; Spada et al. 2013). Hence, a better understanding of pathogen disease ecology in urban stray cat populations, especially in relation to human behavior, is essential for future management attempts to reduce potential epidemiological risks that may occur in both cat and human populations.

## 2 MATERIALS AND METHODS

### 2.1 Study site

This study took place in Seoul, Korea, one of largest megacities in the world with a population density of approximately 17,000/km^2^ and population size 10,290,000 (by year 2016 estimation; Kim & Baik, 2005). Stray cats in the city are most commonly observed in residential areas including apartment complexes and intensive housing areas. Six districts were selected as study sites out of a potential 25 administrative districts in Seoul, based on the results of a nation‐wide survey of cat caretakers performed at the end of 2013 (Kim et al., 2016). All districts within the city were ranked using the synthetic index reflecting the intensity of “cat caretaker activity (hereafter CCA).” The CCA index was based on the following six variables from the survey: (1) proportion of survey respondents per district (who identified themselves as cat caretakers), (2) proportion of respondents who are taking care of more than 10 cats, (3) proportion of respondents who have been working as a cat caretaker for more than 5 years, (4) proportion of respondents who provide food supplement daily in regular manner, (5) proportion of respondents who provide food in areas further than 100 m radius from their house, (6) a score from the subjective perception of each respondent about the intensity of cat caretaking activity in his/her residing district, and two additional demographic factors of the cat caretakers, (7) matriculation rates, and (8) property tax (index of wealth), which were known to be positively associated with the intensity of CCA (Finkler, Hatna, & Terkel, 2011). We performed a principal component analysis for these eight variables of CCA. The first principal component (PC1) explained 54% of the variance in the data, and was used as the CCA index to rank the districts. From this rank, three districts from the top range (high CCA districts representing areas with high supplemental feeding) and three districts at the bottom (low CCA districts representing areas with low supplemental feeding) were selected for this study (Figure S1). All six districts are within the metropolitan area of Seoul city, which is overall extremely urbanized, mostly composed of mixture of commercial and residential areas, and seldom industrial areas (Figure S2).

### 2.2 Sample collection

In each district, selected animal hospitals have annual contracts with their local district government to perform trap‐neuter‐release (TNR) of stray cats. The cats are purposefully trapped within the residential area of each district as the objective of TNR is to reduce the human–cat conflict within these areas (Dr. Jinsun, Bae. personal communication). From the six districts, we collected blood samples of 186 cats during TNR procedures. During TNR procedures, sex and body mass of each cat was recorded. All individuals previously neutered or determined by the veterinarians as underaged (below 1.5 kg) were released without neutering or sampling. Whole blood samples stored in EDTA tubes were used for hematological analysis and for making blood smears for differential counts of white blood cells (WBC). Serum was separated from within 24 hr of blood collection by centrifugation at 1500 rpm for 15 min and stored at −70°C.

### 2.3 Analysis of body condition estimates

Five commonly used biological parameters were used as indices of body condition in this study: body mass (MASS; measured in kg), hematocrit (HCT), serum albumin (ALB), serum creatinine (CREA), and blood urea nitrogen (BUN) from serum (Gilot‐Fromont et al., 2012; Milner et al., 2003). HCT measures the volume percentage of red blood cells in the blood and indicates the aerobic capacity of blood. HCT has been commonly used to assess body condition, and its association with survival, reproduction, and pathogen infection status of host animals has been observed in previous studies (Bókony, Seress, Nagy, Lendvai, & Liker, 2012; Budischak, Jolles, & Ezenwa, 2012). Albumin, creatinine, and blood urea nitrogen are also used as indicators of body condition, providing information on protein supply and metabolism (Milner et al., 2003; Säkkinen et al., 2001). For instance, decreased serum albumin and creatinine concentration can indicate long‐term protein deficiencies, whereas lowered BUN may suggest short‐term protein deprivation (Caldeira, Belo, Santos, Vazques, & Portugal, 2007; Robert & Schwanz, 2013). Hematology and serum biochemical analyses were performed at Seoul National University Veterinary Medical Teaching Hospital (SNU VMTH) using automatic analyzers (hematological analysis: Siemens ADVIA 2120i hematology system; 25 serum biochemistry analysis: Hitachi 7180 clinical analyzer). Blood parameters were interpreted based on the reference range of healthy domestic cats provided by SNU VMTH (Table S1).

### 2.4 Analysis of immune parameters

To capture the complexity of vertebrate immune defenses, we measured seven immune parameters encompassing innate and adaptive defenses. Among WBC differential counts (neutrophils, monocytes, lymphocytes, and eosinophils), neutrophils and monocytes were used as indicators of innate immunity, whereas lymphocytes and eosinophils may reflect both innate and adaptive immune function aspects (Gilot‐Fromont et al., 2012; Young et al., 2016). A leukocyte differential count was performed by counting neutrophils, lymphocytes, eosinophils, or monocytes among 100 leukocytes by examination of air‐dried, whole blood films on a microscope slide stained with Diff‐Quick stain (Medion Diagnostics, Dudingen, Switzerland.). Neutrophil:lymphocyte (N:L ratio) was also calculated as an index of stress in each individual. Based on the effect of corticosteroid stress hormone on reducing number of lymphocyte while increasing neutrophils in multiple vertebrate taxa, N:L ratio is considered a reliable marker for glucocorticoid levels (Davis, Maney, & Maerz, 2008) and has been applied in previous studies as index of stress (Bosson, Islam, & Boonstra, 2012; Johnstone, Lill, & Reina, 2012). We measured two humoral immune parameters: the bacterial killing assay (BKA) and total immunoglobulin G (IgG) concentration as indicators of innate and adaptive immune defenses, respectively (Lee, 2006; Schneeberger, Courtiol, Czirják, & Voigt, 2014). Specifically, BKA against *E. coli* and *S. aureus* characterizes functionally relevant actions of complement proteins and natural antibodies, a reflection of innate immunity (French et al., 2010). We measured bacterial killing activity (hereafter BKA) following the method of French and Neuman‐Lee (2012) with slight modifications. To perform the BKA, commercially standardized pellets of *E. coli* (ATCC No. 8739) and *S. aureus* (ATCC No. 6538; KCCM, Seoul, Korea) were used. Each pathogen pellet was dissolved in 50 ml of sterile 1 mol/L phosphate‐buffered saline (PBS) by vortex. The solution was incubated at 37°C and refrigerated (4°C) for no longer than 24 hr until used as original stock; it was then diluted stepwise from 10^1^ to 10^5^ in order to compare and select the dilution factor to be used for the assay. Log‐phase growth was determined by incubating five different diluent stocks (10^1^ to 10^5^) in 37°C simultaneously with four replicates for each diluent and four negative controls in a 96‐well plates. The absorbance of each diluent was measured before the incubation and after 2, 4, 6, 12, and 24 hr to identify the time‐point at which the absorbance saturated using a spectrophotometer at wavelength 600 nm. For both pathogens, the diluent of 10^3^ showed a clear saturation point at 6 hr and at 12 hr for *E. coli* and *S. aureus*, respectively. For the assay of samples, a 1:8 dilution of each serum samples was mixed with media and diluted pathogen stock, and incubated for 6 and 12 hr in 37°C for *E. coli* and *S. aureus*, respectively. Positive controls containing only media and bacterial solution, and negative controls containing only media, were also included in all tested 96‐well plates. The absorbance was read before the incubation (background absorbance) and after the incubation. The bactericidal ability was calculated by dividing the mean absorbance for each sample (all samples ran in duplicate) by mean absorbance for the positive controls, subtracted from one, which provided the BKA of each sample.

IgG concentration, a measurement of adaptive immunity, reflects the cumulative contact of individual hosts with pathogens and can indicate ongoing pathogen infections (Schneeberger et al., 2014). Total IgG in serum samples was quantified using a commercialized sandwich ELISA kit (Abnova IgG (Cat) ELISA‐Abnova, Taipei City, Taiwan), standardized for domestic cats, following protocols provided by the manufacturer. Absorbance of the ELISA kit was quantified by reading the optical density (OD) of the reaction plate using a microplate reader (Biotek, Winooski, VT, U.S.A.) at wavelength 450 nm.

### 2.5 Statistical analysis

**Figure 1:**
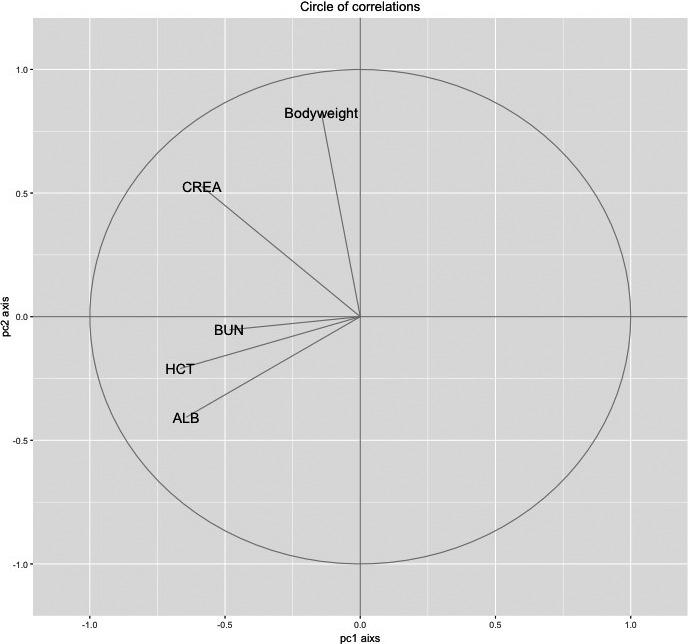
Correlation circle showing the projection of body condition parameters on principal components. See text for definition of variable (HCT: hematocrit, BUN: blood urea nitrogen, ALB: albumin, CREA: creatinine)

We collected 186 samples for the study, 83 from high and 103 from low CCA districts, respectively. Among them, one individual from a low CCA district showed extremely high BUN (52.9 mg/dl) and CREA (2.59 mg/dl) values, indicative of chronic renal failure, and this influential outlier was removed from all analyses. All statistical analyses were performed using the statistical software R (http://cran.r-project.org). Sex ratio associations of samples between high and low CCA districts were analyzed by chi‐squared tests. We tested for associations among body condition, immune phenotype, and CCA. First, we performed principal component analysis (PCA) on all of the five body condition estimates (body mass (MASS), hematocrit (HCT), serum albumin (ALB), blood urea nitrogen (BUN), and serum creatinine (CREA); Gilot‐Fromont et al., 2012). The first component of PCA captured 25.6% of the total inertia, showing positive correlation with all tested body condition parameters (Figure 1). Thus, PC1 from PCA of body condition estimates were used as a body condition index for further analysis. Immune parameters were not combined as a single index because statistically, when the parameters were analyzed using PCA, a subset of immune parameters correlated in the opposite direction with regard to PC1 or PC2, and could not be represented by a single index. Next, to test for evidence of association of body condition index and immune parameters with CCA and sex, we performed a linear mixed model (LMM). Separate models were built for each response variable (body condition index, N:L ratio, and each immune parameter), and all models were set with CCA, sex, and its interaction as fixed variables and individual district as a random variable. Transformation was applied to response variables when required to fulfill model normality assumptions of LMM. Square root, logarithmic, and square power transformation were applied to values of BKA‐*E. coli*, N:L ratio, and IgG concentration, respectively. Owing to the skewed distribution of the count of each WBC (neutrophil, monocyte, lymphocyte, and eosinophil), the association between CCA and sex with neutrophils and lymphocytes was analyzed through GLMM, fitted using the penalized quasi‐likelihood approach, whereas eosinophils and monocytes were analyzed by negative binomial GLMM. Wald chi‐squared tests were used to determine the effect size of estimated parameters in the (G)LMM. All the *p*‐values from the output of mixed models were corrected through Benjamini–Hochberg procedure which controls the false discovery rate.

## 3 RESULTS

We analyzed 185 individuals, 83 and 102 from high and low CCA districts, respectively. The body condition index (PC1 from PCA of body condition estimates) was significantly higher in males (Wald χ^2^ = 8.82, *p* < .01), but sex ratio did not differ between high and low CCA sites (chi‐squared test; *p* = .76). Body condition index showed no clear association with CCA (*p* = .88) or immune phenotype estimates (neutrophils, monocytes, lymphocytes, and eosinophils counts, IgG concentration, BKA‐*E. coli* and BKA‐*S. aureus*).

**Figure 2:**
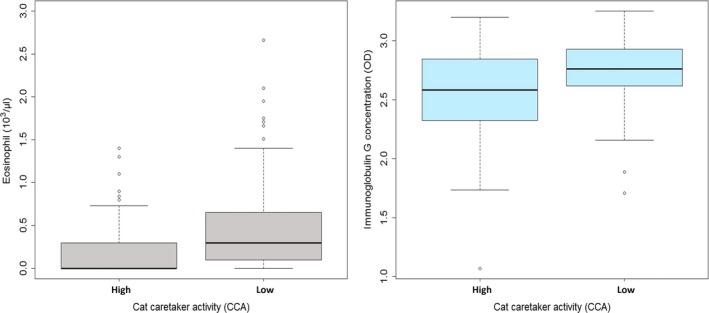
Box plot of IgG concentration and Eosinophil counts by cat caretaker activity

**Figure 3:**
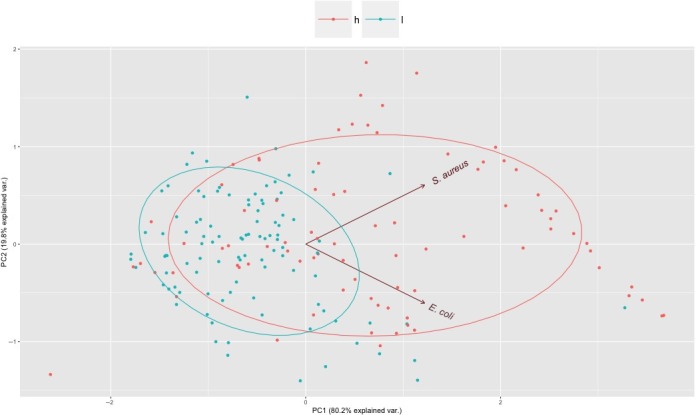
Bacterial killing assay (BKA) results between two cat caretaker activity districts (h = High, l = Low). Projections of BKA value of all studied individuals on the first two principal components of PCA simultaneously analyzing BKA for *E. coli* and *S. aureus* (displayed in the PC1‐PC2 plane)

Each immune parameter was separately analyzed to investigate its association with CCA, sex, and its interactions. Among WBC components (neutrophils, monocytes, lymphocytes, and eosinophils), none of the blood cell types showed significant differences between high and low CCA populations except for eosinophils. Cats from low CCA districts showed slightly higher eosinophil cell counts than high CCA districts (Wald χ^2^ = 5.09, *p* = .07; Figure 2). Similarly, IgG concentration was significantly higher in low CCA districts (Wald χ^2^ = 20.97, *p* < .01; Figure 2, Table 1). Conversely, BKA of *E. coli* and *S. aureus* (BKA‐*E. coli*; Wald χ^2^ = 15.77, *p* < .01, BKA‐*S. aureus*; Wald χ^2^ = 31.89, *p* < .01) was significantly higher in cats from high CCA districts (Figure 3, Table 1).

**Table 1:** Result of LMM, GLMM association analysis of immune parameters by cat caretaker activity (CCA) and sex

Parameters	Variable	Estimate	*SE*	Lower confidence interval	Upper confidence interval	*Z* (*t*) value	*p* Value
BKA‐*E. coli*	CCA‐high	0.14	0.05	0.04	0.25	2.68	.00^a^
BKA‐*S. aureus*		0.24	0.07	0.10	0.38	3.40	.00^a^
IgG		−1.09	0.34	−1.75	−0.42	−3.22	.00^a^
Eosinophil		−0.86	0.48	−1.88	0.18	−1.78	.07
BKA‐*E. coli*	Sex‐female	0.04	0.05	−0.05	0.13	0.78	.14
BKA‐*S. aureus*		0.01	0.05	−0.09	0.11	0.18	.68
IgG		−0.43	0.31	−1.04	0.18	−1.37	.14
Eosinophil		−0.18	0.28	−0.55	0.52	−0.07	.87

a *p*‐Values below .00.

In relation to sex, tested immune parameters showed no significant variation between female and male. The N:L ratio did not show significant association with CCA or sex (CCA; Wald χ^2^ = 0.00, *p* = .99, Sex; Wald χ^2^ = 0.38, *p* = .99). Result of GLM and GLMM analysis for association between CCA and sex with rest of the variables is presented in Table S2.

## 4 DISCUSSION

The goal of this study was to examine how human food provisioning is associated with body condition and immune phenotype of stray cats. Unlike our initial hypothesis, cat caretaker activity (CCA) did not show a significant association with body condition indices. However, our results suggest that different aspects of immunity can vary in association with supplemental feeding. Stray cats from high intensity CCA sites had evidence of higher innate defense (BKA) but significantly lower adaptive defense (IgG concentration) compared to cats from low CCA areas. Additionally, eosinophil counts, which help the immune system to fight multicellular parasites such as helminth and ectoparasites, were also lower in cats from high CCA sites. These findings reflect potential impacts of supplemental food on the immune phenotype of animals through routes somewhat unrelated to overall body condition.

Constitutive innate immune responses, such as BKA, are sensitive to energy availability (Lee, 2006). Previous studies reported direct trade‐offs between innate immune responses and other energy‐demanding physiological processes, and that these trade‐offs were relieved by food supplementation (Brzek & Konarzewski, 2007; French & Moore, 2008). In general, animals in better condition are believed to maintain higher constitutive innate immune function (Lee, 2006). In this study, BKA for both bacteria (*E. coli* and *S. aureus*) was higher in high CCA districts and lower in low CCA districts, which may be explained by different availability of a stable food source in each area. For instance, food provisioning by cat caretakers can allow cats to spend less time and energy foraging and/or competing to secure food sources, leaving more energy for other physiological processes, including maintenance of innate immunity (Becker et al., 2015; Lane, Holley, Hollocher, & Fuentes, 2011). Alternatively, higher BKA may have been related to the nutritional quality of the food source available. In addition to proteins, micronutrients, such as vitamin and mineral, are increasingly recognized as critical components of innate immune responses (Chen et al. 2014; Ruiz et al., 2010). Stray cats in the low CCA area, such as other urban wildlife, will commonly rely on indirectly provisioned human food sources from garbage or dumpsters. These food sources are unlikely to contain nutritional elements critical for maintaining essential immune components for the cats, such as vitamins, and/or antioxidants (Koski & Scott, 2001; Maggini, Wintergerst, Beveridge, & Hornig, 2007; Marcos, Nova, & Montero, 2003). In comparison, the cats in the high CCA districts are strictly supplemented with commercial cat food by caretakers, allowing them to maintain a relatively balanced nutritional status, potentially giving them advantage in eliciting stronger innate immune response, such as bactericidal ability.

However, the abundance of energy availability due to supplemental feeding is limited in explaining our observation of higher IgG concentrations in lower CCA areas. IgG is predominantly involved in the secondary (acquired) immune response to pathogens. One explanation may be related to the fact that the assay used in this study measured concentration of cumulative IgG rather than an instantaneous antibody response. Therefore, the result of IgG concentrations observed in this study may reflect the animal's history of pathogen infection, which may be affected by factors such as repetitiveness, duration, and frequency of pathogen exposure (Brock, Hall, Goodman, Cruz, & Acevedo‐Whitehouse, 2013; Listi et al. 2006). Similarly, previous studies interpreted increased IgG concentration as a sign of chronic infection or accumulation of repeated pathogen exposure (Brock et al., 2013; Schneeberger et al., 2014). Although extreme malnutrition may hinder production of IgG (Frouin, Haulena, Akhurst, Raverty, & Ross, 2013; Glick, Day, & Thompson, 1981), this is less likely to apply in our study, as overall cats showed similarly moderate body condition regardless of the CCA intensity. Hence, our observation of higher IgG levels in low CCA areas may be better explained by the difference in pathogen exposure in the high and low CCA areas with cats in the low CCA areas having higher exposure to a wide variety of pathogens. Supplemental feeding in wildlife is known to shift animal behavior in ways that can affect the exposure and transmission of pathogens among hosts (Murray, Becker, Hall, & Hernandez, 2016). For instance, smaller home range areas and/or less time spent foraging (Gilchrist & Otali, 2002; Lemel, Truve, Soderberg, & Soederberg, 2003; Schoepf, Schmohl, König, Pillay, & Schradin, 2015) are often reported in animals provided with abundant supplemental food. Such altered behavior and/or habitat use can potentially lower the exposure of animals to environmentally transmitted pathogens (Fredebaugh, Mateus‐Pinilla, McAllister, Warner, & Weng, 2011; Parr, Fedigan, & Kutz, 2013). Similarly, stray cats in low CCA districts are expected to use larger home ranges and/or spend more time seeking food sources, giving them greater chances to be exposed to infectious stages of pathogens or parasites in the environment. Another possible explanation worth further exploring is the role of neutering in altering the behavior and physiology of cats, with cascading effects on pathogen interaction and immune phenotype. Two of three high CCA sites from this study were the most affluent neighborhoods within the city, and numerous cat caretakers from these sites are well‐known for systematically neutering cats within their neighborhood without the help of funds from the city for almost a decade (Dr. Jinsun, Bae. personal communication). A higher proportion of neutered cats in the area may contribute to relieving stress of intact cats from breeding competition that may lead to altered energy distribution, such as increased availability of energy to invest in immunity (Martin et al., 2008). Therefore, in the case of animals where neutering is performed as in stray cats, its additional impact on overall stress, immune response, and body condition will need to be considered simultaneously.

The observed pattern of eosinophil counts may be also explained within the context of increased environmentally transmitted pathogen exposure, such as helminths (Klion & Nutman, 2004), in cats from low CCA sites. Stray cats are hosts of a suite of helminths, such as hookworms and protozoa, such as *Toxocara* sp. Many of these parasite are transmitted through environmental contact such as soil and fecal matter (Borji et al., 2011; Waap, Gomes, & Nunes, 2014). Thus, higher eosinophil counts in low CCA districts may be the result of higher helminth worm burden of cats in the areas, potentially acquired through environmental transmission while exploring for food. A lack of supplemental food in lower CCA districts may have contributed to more active foraging and/or intensive land use by stray cats, facilitating repeated and/or frequent contact with infectious stages of pathogens followed by higher IgG concentration and/or eosinophil count. Another potential underlying mechanism may be associated with higher population density of cats in lower CCA districts compared to high CCA districts (JH unpublished data), as high prevalence of environmentally transmitted pathogens has been frequently related to high host population density (Arneberg et al. 1998).

Lastly, the lower BKA observed in low CCA sites may be explained by high energy expenditure of these cats in response to frequent exposure to certain pathogens, such as viruses or helminths (McDade, Georgiev, & Kuzawa, 2016; Palacios, Cunnick, Winkler, & Vleck, 2012). Similar energetic trade‐offs among different immune components have been discussed and observed in previous studies (Martin, Weil, Kuhlman, & Nelson, 2006; Pigeon, Bélisle, Garant, Cohen, & Pelletier, 2013). For instance, in a study of different bat colonies, bats in areas potentially exposed to fungi and parasites in higher rate, hence requiring stronger memory‐related resistance, showed higher T‐cell‐mediated immune responses while their bactericidal ability was lower, showing negative correlation between the two immune parameters (Allen et al., 2009; Brock et al., 2013; Schneeberger et al., 2014).

The potential influence of human supplemental feeding on interactions between animals and their pathogens and parasites occurs through various routes, such as altered animal behavior or immune function (Becker et al., 2015; Murray et al., 2016). Here, we provide empirical data on the relationship between food provisioning and immune phenotype and discussed how under the influence of artificial food sources, immune phenotype and host–pathogen interactions may mutually affect each other. Our results demonstrate that different immune parameters show associations with supplemental feeding in nonuniform directions. While energy costly immune responses may better reflect the availability of supplemental food, as in BKA from this study, rates of other immune components may be a better indicator of other biological processes, such as pathogen exposure history, which may explain the IgG results from this study. The influence of supplemental feeding on the immune system is likely to go beyond the energetic‐immune response relationship, following different scenarios depending on the diverse behavioral and physiological responses of the animals that are supplementary fed (Murray et al., 2016). Such responses may be further complicated by the different types of food provisioning (e.g., tourist site, garbage, caretaker) which may vary in its characteristics including duration, regularity, nutritional value of food sources, which call for further study regarding its physiological and epidemiological impacts.

Supplemental feeding is a widely practiced activity with various purposes, from a conservation management strategy, to feeding as tourist attraction, sometimes with unintended consequences (Semeniuk, Bourgeon, Smith, & Rothley, 2009). In order to have a better understanding of how anthropogenic food supplementation is altering not only immune function, but also infectious disease transmission and wildlife health, future research may benefit by simultaneously evaluating multiple physiological and behavioral parameters in wildlife populations.

## CONFLICT OF INTEREST

None declared.

## AUTHOR CONTRIBUTIONS

JH and NG wrote the paper; JH, HL, and NG designed the study; JH, NG,and YK analyzed the data; JH, YK, SL, NK, MC performed laboratory analyses.

## DATA ACCESSIBILITY

Data available from the Dryad Digital Repository: data not submitted yet.
