# Supplementary figures and images for: Anthropogenic food provisioning and immune phenotype: Association among supplemental food, body condition, and immunological parameters in urban environments
Source: Ecol Evol. 2018 Feb 17;8(5):3037–46. doi: 10.1002/ece3.3814 (PMC5838038; doi:10.1002/ece3.3814)

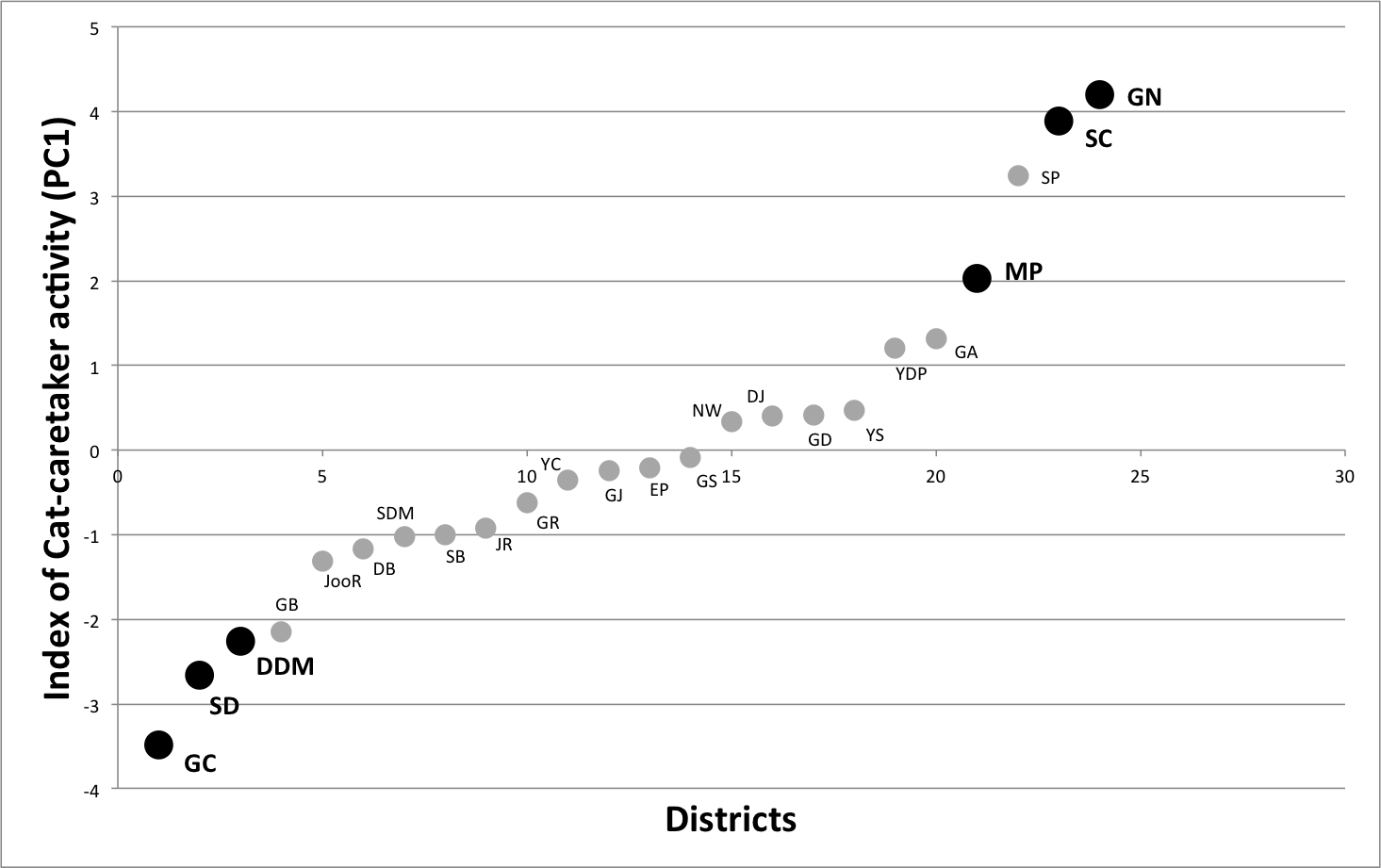

Supplement: Supplementary file 1 [file ECE3-8-3037-s001.png]
