# Supplementary material for: Anthropogenic food provisioning and immune phenotype: Association among supplemental food, body condition, and immunological parameters in urban environments
Source: Ecol Evol. 2018 Feb 17;8(5):3037–46. doi: 10.1002/ece3.3814 (PMC5838038; doi:10.1002/ece3.3814)
Supplement: Supplementary file 2 [file ECE3-8-3037-s002.docx]

**Figure S2.** Land-use map and coordinates of six districts within Seoul, Korea where the study samples were collected

High CCA sites

Gangnam district (GN): 37°31'2.13"N, 127° 2'50.37"E

Seocho district (SC): 37°29'1.36"N, 127° 1'56.68"E

Mapo district (MP): 37°33'49.52"N, 126°54'30.32"E

Low CCA sites

Dongdaemun district (DDM): 37°34'27.73"N, 127° 2'24.07"E

Geumcheon district (GC): 37°27'6.67"N, 126°54'7.33"E

Seongdong district (SD): 37°33'48.03"N, 127° 2'13.57"E
