# Supplementary material for: Anthropogenic food provisioning and immune phenotype: Association among supplemental food, body condition, and immunological parameters in urban environments
Source: Ecol Evol. 2018 Feb 17;8(5):3037–46. doi: 10.1002/ece3.3814 (PMC5838038; doi:10.1002/ece3.3814)
Supplement: Supplementary file 3 [file ECE3-8-3037-s003.docx]

|  | **Reference range** | **High CCA** | | **Low CCA** | | |
| --- | --- | --- | --- | --- | --- | --- |
|  |  | **Male**  **(n=41)** | **Female**  **(n=42)** | | **Male**  **(n=47)** | **Female**  **(n=55)** |
| **Body mass (kg)** | - | 4.54  (4.19-4.90) | 3.96  (3.69-4.23) | | 4.16  (3.85-4.47) | 3.39  (3.20-3.58) |
| **HCT**  **(%)** | 27.7-46.8 | 30.48  (27.75-33.30) | 28.74  (27.04-30.44) | | 30.36  (28.84-31.88) | 28.85  (27.60-30.10) |
| **RBC (x10^6^/L)** | 6.0-10.1 | 6.74  (6.14-7.33) | 6.43  (6.04-6.83) | | 6.68  (6.33-7.03) | 6.22  (5.93-6.53) |
| **ALB (g/dL)** | 8.1-14.2 | 2.92  (2.81-3.02) | 2.74  (2.57-2.91) | | 2.88  (2.80-2.98) | 2.94  (2.88-2.99) |
| **BUN (**mg/dL**)** | 41.3-52.6 | 19.34  (18.15-20.52) | 18.59  (17.43-19.75) | | 17.97  (16.94-18.99) | 17.23  (16.13-18.32) |
| **CREA (**mg/dL**)** | 6.3-19.6 | 1.04  (0.99-1.10) | 0.97  (0.92-1.03) | | 1.07  (1.00-1.13) | 1.03  (0.97-1.08) |

**Table S1.** Hematological parameter values and body mass by CCA and sex
