# Supplementary material for: Anthropogenic food provisioning and immune phenotype: Association among supplemental food, body condition, and immunological parameters in urban environments
Source: Ecol Evol. 2018 Feb 17;8(5):3037–46. doi: 10.1002/ece3.3814 (PMC5838038; doi:10.1002/ece3.3814)
Supplement: Supplementary file 4 [file ECE3-8-3037-s004.docx]

**Table S2. Complete result of (G)LMM association analysis of immune and body condition parameters by CCA and sex**

| **Parameters** | **Variable** | **Estimate** | **SE** | **Lower confidence interval** | **Upper confidence interval** | **Z (t) value** | **P value*** |
| --- | --- | --- | --- | --- | --- | --- | --- |
| **Body condition index** | **CCA-high** | 0.18 | 0.21 | -0.25 | 0.60 | 0.87 | 0.91 |
| **Body weight** |  | 0.38 | 0.24 | -0.12 | 0.89 | 1.61 | 0.27 |
| **Hematocrit** |  | -0.02 | 0.02 | -0.07 | 0.03 | -0.65 | 0.79 |
| **Albumin** |  | 1.20 | 1.53 | -1.83 | 4.22 | 0.78 | 0.42 |
| **BUN** |  | 0.07 | 0.04 | -0.00 | 0.14 | 1.74 | 0.03 |
| **Creatinine** |  | -0.02 | 0.02 | -0.08 | 0.03 | -0.71 | 0.31 |
| **Neutrophil** |  | -0.03 | 0.06 | -0.15 | 0.10 | -0.55 | 0.91 |
| **Lymphocyte** |  | -0.17 | 0.16 | -0.60 | 0.26 | -1.09 | 0.60 |
| **Monocyte** |  | -0.44 | 1.75 | -4.36 | 3.28 | -0.25 | 0.92 |
| **N:L ratio** |  | 0.03 | 0.21 | -0.40 | 0.54 | 0.16 | 0.99 |
| **Body condition index** | **Sex-female** | -0.30 | 0.20 | -0.68 | 0.10 | -1.51 | 0.00 |
| **Body weight** |  | -0.77 | 0.18 | -1.13 | -0.41 | -4.24 | 0.00 |
| **Hematocrit** |  | -0.02 | 0.02 | -0.06 | 0.01 | -1.35 | 0.34 |
| **Albumin** |  | 1.18 | 1.43 | -1.63 | 3.99 | 0.83 | 0.42 |
| **BUN** |  | -0.04 | 0.04 | -0.11 | 0.03 | -1.12 | 0.21 |
| **Creatinine** |  | -0.02 | 0.02 | -0.06 | 0.02 | -1.14 | 0.13 |
| **Neutrophil** |  | -0.01 | 0.04 | -0.09 | 0.08 | -0.18 | 0.81 |
| **Lymphocyte** |  | 0.02 | 0.13 | -0.23 | 0.27 | 0.16 | 0.60 |
| **Monocyte** |  | -1.74 | 1.16 | -4.03 | -0.55 | -1.50 | 0.10 |
| **N:L ratio** |  | -0.05 | 0.17 | -0.38 | 0.28 | -0.32 | 0.99 |

*P values are corrected for multiple testing through Benjamini-Hochberg procedure
